# Geometry-informed correction of projection bias in browser-based monocular squat assessment

**DOI:** 10.3389/fspor.2026.1831625

**Published:** 2026-06-25

**Authors:** Ryota Iizuka, Koki Yamada, Mizuki Sato, Naka Gotoda, Ryota Akagi

**Affiliations:** 1 Graduate School of Engineering and Science, Shibaura Institute of Technology, Saitama-shi, Saitama, Japan; 2 Integrated Center for Informatics, Kagawa University, Takamatsu-shi, Kagawa, Japan; 3 College of Systems Engineering and Science, Shibaura Institute of Technology, Saitama-shi, Saitama, Japan

**Keywords:** geometric correction, markerless motion capture, monocular pose estimation, projection bias, rehabilitation monitoring, squat biomechanics

## Abstract

**Introduction:**

Monocular RGB-based human pose estimation is increasingly applied in field-based movement assessments; however, systematic projection-related errors inherent to the simplified camera geometry remain insufficiently characterized relative to laboratory-based biomechanical standards. This study quantified systematic projection bias in a browser-based markerless motion capture (MMC) system and validated a geometry-informed linear correction framework for sagittal-plane squat analysis under standardized monocular acquisition conditions.

**Methods:**

Bodyweight squats performed by 30 healthy adult males were simultaneously recorded using a three-dimensional marker-based optical motion capture (OMC) system and a two-dimensional webcam positioned at a fixed distance and orientation.

**Results:**

Raw joint angles obtained from the MMC system revealed significant systematic underestimation (hip: −11.2°, knee: −10.6°), consistent with perspective-induced projection effects predicted by the pinhole camera model and differences in the joint center definitions. To address this projection-consistent bias, a geometry-constrained linear correction model was developed and validated using leave-one-subject-out cross-validation. The correction effectively neutralized the systematic bias (hip: 0°, *p* = 0.323; knee: 0°, *p* = 0.645) and substantially reduced root mean square error (hip: from 11.6° ± 4.2° to 4.0° ± 2.3°; knee: from 10.9° ± 3.3° to 3.9° ± 1.5°). Furthermore, mean coefficients of determination were significantly improved and stabilized (hip: from 0.31 ± 0.94 to 0.90 ± 0.17; knee: from 0.67 ± 0.30 to 0.96 ± 0.04).

**Discussion:**

Importantly, high accuracy was achieved without incorporating participant-specific anthropometric variables, suggesting that projection-consistent geometric factors predominated under controlled camera conditions. These findings demonstrate that systematic errors in monocular pose estimation can be substantially mitigated when the acquisition distance and orientation are standardized. Our results suggest that under such predefined recording constraints, corrected joint angles provide practically relevant estimates for strength assessment and rehabilitation monitoring.

## 1 Introduction

Squatting is one of the most fundamental exercises in strength training, rehabilitation, and sports science, targeting multiple muscle groups in the lower extremities and core (1–3). Dynamic squats primarily strengthen the hip, thigh, and back muscles, which are critical for running, jumping, and lifting (4). Accurate monitoring of joint angles, particularly hip and knee flexion-extension in the sagittal plane, is crucial to ensure exercise efficacy and prevent musculoskeletal injuries (5, 6). Although three-dimensional (3D) marker-based optical motion capture (OMC) systems remain the gold standard for kinematic analysis, offering sub-millimeter precision (7), their high cost, complexity, and requirement for controlled laboratory environments limit their feasibility for home-based training and clinical telerehabilitation (8, 9).

Recent advancements in computer vision have led to the development of markerless motion capture (MMC) technologies capable of estimating human kinematics from a standard two-dimensional (2D) RGB video (10–12). Among these, MoveNet has been adopted for real-time browser-based applications. The Lightning variant provides a computationally efficient architecture suitable for execution via TensorFlow.js on consumer-grade devices. Despite this feasibility, the use of monocular-webcam-based MMC systems in clinical or high-performance settings is limited by concerns about their measurement accuracy.

Previous investigations have reported systematic discrepancies between monocular 2D pose estimation and 3D OMC systems, particularly during high-range-of-motion tasks such as deep squats (13–16). Importantly, these discrepancies are not purely random but are largely attributable to projection-consistent geometric effects inherent to monocular imaging. In the pinhole camera model (17), three-dimensional limb segments that rotate out of the image plane undergo perspective-induced foreshortening when projected onto a two-dimensional sensor. For example, during deep squat descent, the thigh segment rotates considerably out of the sagittal plane (hip abduction/rotation), forming an oblique angle *θ* relative to camera sensor's plane. As a result, the projected 2D length of the segment is reduced by a factor proportional to cos(*θ*), causing systematic underestimation of the knee flexion angle as the squat depth increases.

Such projection-consistent bias is further amplified when the camera alignment deviates from the midsagittal plane or when participants exhibit minor transverse-plane rotations (e.g., 5°–10°). In addition, the variation in the camera-to-subject distance introduces differential perspective distortion, whereby proximal segments may appear disproportionately larger than distal segments (11). Because these effects arise from deterministic geometric principles rather than stochastic noise, they are theoretically predictable under standardized acquisition conditions.

Highly accurate markerless solutions that use multicamera arrays are already being used in commercial and clinical environments. However, these systems often rely on complex, high-cost, and non-portable setups that remain inaccessible for typical home-based use. Furthermore, although research has investigated lifting 2D poses to 3D using complex neural networks (18, 19) or multicamera calibration (16, 20), these approaches primarily seek to reconstruct the spatial depth rather than to explicitly characterize and model projection-induced bias under constrained monocular conditions. Consequently, the question remains whether a systematic error observed in the simplified browser-based configurations can be mathematically characterized and mitigated without resorting to complex 3D reconstruction pipelines. If projection-related discrepancies are geometrically consistent, fixing the acquisition geometry may allow for systematic bias to be isolated and corrected through transparent mathematical modeling. Such an approach would provide an interpretable alternative to data-intensive correction strategies while preserving the practical advantages of low-cost monocular systems.

Therefore, this study aimed to characterize projection-consistent bias in browser-based monocular squat assessment and to determine whether this bias can be systematically mitigated under standardized camera geometry. Specifically, our objective was to 1) quantitatively characterize projection-related discrepancies between the MoveNet SinglePose Lightning system and a 3D OMC reference during sagittal plane squats and 2) develop and validate a geometry-constrained linear correction model under fixed acquisition conditions. We hypothesized that, when the camera distance and orientation were standardized, projection-induced errors would exhibit stable linear characteristics that permitted clinically acceptable correction without incorporating participant-specific anthropometric variables. By clarifying the deterministic geometric basis of the measurement error, this study sought to establish a standardized framework for condition-aware correction in low-cost browser-based biomechanical assessment.

## 2 Methods

### 2.1 System development

**Figure 1:**
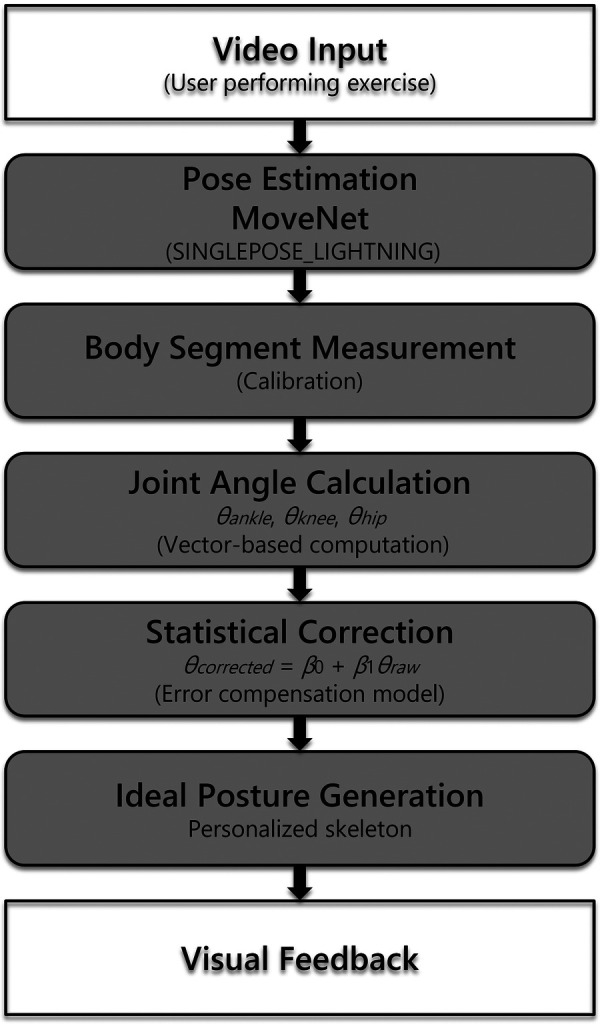
Overall pipelines of the browser-based data acquisition system and linear correction framework.

To enable accessible home-based monitoring, we implemented a browser-based data acquisition system and developed a validated linear correction framework for post processing. The overall architecture of the developed system is illustrated in Figure 1. The process consists of the following stages:

(a) Video Input: Captures real-time sagittal plane movement using a standard consumer-grade webcam.
(b) Pose Estimation (MoveNet): Uses a lightweight, bottom-up CNN architecture to localize 17 body keypoints via heat maps and offset regression.
(c) Body Segment Measurement: Automatically estimates the lengths of segments (e.g., femur and shank) based on the distance between the predicted 2D keypoint coordinates.
(d) Joint Angle Calculation: Computes sagittal plane joint angles for the hip and knee using vectors derived from the keypoint positions.
(e) Statistical Correction: Applies the validated linear regression model to the raw kinematic data to compensate for systematic underestimation caused by perspective distortion and joint center mismatch.
(f) Ideal Posture Generation: Compares the corrected joint angles with user-defined target values (e.g., target hip angle) to generate reference benchmarks for correct form.
(g) Visual Feedback: Delivers high-precision real-time metrics and alerts directly to the user interface via a privacy conscious browser (TensorFlow.js).

#### 2.1.1 Architecture and pose estimation

The system used the MoveNet SinglePose Lightning model, a lightweight bottom-up deep-learning model optimized for high-speed inference on resource-constrained devices (21). The model used a MobileNetV2 feature extractor (22) and a prediction scheme inspired by CenterNet (23) to predict human keypoints densely, eliminating the need for computationally expensive non-maximum suppression. The application was built to operate entirely on the client side using TensorFlow.js, ensuring user privacy by processing video feeds locally within the browser, without uploading raw data to external servers. To provide continuous and smooth joint angle calculations, especially during full extension (near 180°), we implemented calculation logic using the four-quadrant inverse tangent function (atan2) based on the extracted keypoint coordinates, thereby eliminating the computational discontinuities associated with conventional inverse cosine functions.

#### 2.1.2 Hardware requirements

A standard consumer-grade webcam with a VGA resolution (640 × 480 pixels) was selected for its affordability and widespread availability (11). This choice enables home-based deployment as it requires no specialized hardware beyond a typical laptop. The system is designed to achieve real-time performance (typically more than 30 fps) on modern devices, providing a sufficient temporal resolution to track dynamic movements such as squats (21).

#### 2.1.3 Real-time feedback and visualization

A real-time visual feedback system was implemented using the p5.js library to standardize movement execution and ensure consistent kinematic data collection. The system begins with a 10-s initialization phase to estimate participant-specific segment lengths from a standing neutral pose. During squat execution, the interface overlays the estimated pose and dynamically adjusts the color of the reference lines to encourage compliance with the target depth, specifically defined as 120° of hip and knee flexion. This feedback mechanism regulates participant movement within the sagittal plane, ensuring that the range of motion is sufficient for a valid comparison with the gold-standard OMC system.

#### 2.1.4 Linear correction framework

After data collection, a *post-hoc* linear correction model was applied to systematically remove perspective-induced errors and labeling-defined biases from the raw joint angle estimates (16). Validation against OMC has shown that single-camera pose estimation models often exhibit systematic offsets and proportional errors, particularly due to limb foreshortening when limbs move at varying depths relative to the camera center (11, 24). Based on the feasibility of regression-based kinematic estimation demonstrated in large-scale clinical video analyses (25), a simple linear regression model $\left(\theta_{\mathrm{corrected}}=\beta_{0}+\beta_{1}\theta_{\mathrm{raw}}\right)$ that captures the collective regression trend observed across the participant group was used; this model was chosen to ensure a low-resource profile suitable for browser-based environments. Although this correction was validated in this study through offline processing, the minimal computational overhead of the two-coefficient model allowed straightforward integration into real-time browser-based systems for future deployment (21). A proof-of-concept browser implementation with real-time correction was developed. Preliminary tests on consumer-grade devices confirmed that the integrated correction model maintains a high processing speed (over 30 fps), demonstrating its feasibility for real-world deployment; however, a formal performance evaluation of the computational load is beyond the scope of this study.

### 2.2 Participants

Thirty healthy male adults participated in this study [age: 23 ± 3 years; height: 1.72 ± 0.07 m; weight: 64.8 ± 12.3 kg; body mass index (BMI): 21.9 ± 3.9 kg/m2]. None of the participants had cardiovascular or musculoskeletal disorders that could impair the squat performance. All participants provided written informed consent, and the study protocol was approved by the Ethics Committee of Shibaura Institute of Technology (Approval No. 23-017).

### 2.3 Experimental protocol

Participants performed 10 continuous repetitions of bodyweight squats paced by a metronome at 60 bpm (3 s each for the descent and ascent phases). This paced protocol ensured the controlled movement speed and minimized the influence of acceleration-induced artifacts on kinematic estimates. To minimize keypoint and marker occlusion by the upper extremities, participants were instructed to keep their arms crossed over their chest throughout the trials.

#### 2.3.1 Reference system

A 3D OMC system (OptiTrack Flex 3, Acuity Inc.) with six infrared cameras served as the ground truth for kinematic validation. Data were recorded at a constant sampling rate of 100 Hz within a 5.0 × 4.0 m capture volume. Passive reflective markers were attached to standardized anatomical landmarks on the right side of the body, including the acromion, greater trochanter, lateral femoral epicondyle, and lateral malleolus, defining the trunk, thigh, and shank segments. Before each session, the system was calibrated using a dedicated wand (CW-500, Acuity Inc.), achieving a mean spatial reconstruction error of 0.10 ± 0.01 mm. The global coordinate system was defined using a calibration square (CS-200; Acuity Inc.). To enable direct comparison with 2D markerless data, the reconstructed 3D marker coordinates were projected onto the sagittal plane, with only the anteroposterior and vertical components extracted for kinematic analysis. This projection enables a geometrically consistent comparison with the 2D markerless estimates under the assumption that out-of-plane motion is minimal and symmetrically distributed (14, 26).

#### 2.3.2 Target system

A standard webcam integrated into a laptop PC (OMEN, HP Inc.) was positioned 2.00 m to the right of the participant to capture the sagittal view. The camera height was set at approximately 0.975 m, approximating the average height of participant's greater trochanter to minimize perspective distortion of the hip and knee joints during the squatting motion. This height was selected to simulate the typical placement of a laptop camera, using which a user monitors their own form on the screen. For pose estimation, the MoveNet Lightning model implemented with TensorFlow.js was used (21). This lightweight architecture was chosen to prioritize the real-time inference and low-latency feedback over the raw pixel-level accuracy, providing an immediate monitoring environment within a web browser (Google Chrome). Because pose estimation was performed on the client side, the sampling frequency was inherently variable, depending on browser-rendering updates and CPU/GPU processing loads.

### 2.4 Data processing

#### 2.4.1 Kinematic calculation

Sagittal plane joint angles of the hip and knee were calculated using vector arithmetic. For the OMC system, 3D marker coordinates were projected onto the sagittal plane (*y*–*z* plane) to ensure geometric comparability with the 2D MMC output. The hip angle was defined as the angle between the trunk (acromion to greater trochanter) and thigh (greater trochanter to lateral femoral epicondyle) segments. The knee angle was defined as the angle between the thigh and shank (lateral femoral epicondyle to lateral malleolus) segments.

#### 2.4.2 Synchronization and resampling

Owing to the variable frame rate of the webcam-based system, MMC data were up-sampled to 100 Hz using linear interpolation to match the constant sampling rate of the OMC system. Temporal synchronization was achieved by calculating the cross correlation of the knee flexion angle profiles and applying a time shift to the MMC signal to maximize the correlation coefficient. We considered the knee flexion angle to be the most reliable synchronization signal because it robustly reflects the movement phases during a squat task. This procedure was performed independently for both hip and knee joint signals to account for potential intersegmental latency in the pose estimation algorithm. After resampling, all kinematic data across the cohort (*N* = 30) were analyzed, revealing an overall mean sampling frequency of 69.7 ± 1.5 Hz for the initial MMC capture.

#### 2.4.3 Corrections models and their validation

To address projection-consistent systematic errors, three linear regression models were evaluated to map the uncorrected (raw) estimated angles $\left(\theta_{\mathrm{MMC}}\right)$ to the reference angles $\;(\theta_{\mathrm{OMC}})$. Linear regression models were intentionally chosen over more complex nonlinear approaches (e.g., neural networks or high-order polynomials) for three main reasons. First, linear models offer inherent interpretability, which is essential for clinical applications, where understanding the source of error correction is critical (27). Second, given the limited sample size (*N* = 30), simpler models are more robust against overfitting than high-variance complex models. Finally, the low computational cost of linear equations ensures seamless real-time execution in browser-based environments. In addition to the theoretical advantages of interpretability and robustness, a preliminary visual inspection of the uncorrected data indicated that the dominant errors exhibited strong linear trends with respect to the joint angle magnitude. This empirical observation further supported the suitability of the first-order model. Accordingly, the following three linear models were evaluated:

Linear Model:

$$\theta_{\mathrm{OMC}}=\beta_{0}+\beta_{1}\theta_{\mathrm{MMC}}$$

Linear + Anthro Model:

$$\theta_{\mathrm{OMC}}=\beta_{0}+\beta_{1}\theta_{\mathrm{MMC}}+\beta_{2}\mathrm{Height}+\beta_{3}\mathrm{Weight}$$

Linear + BMI Model:

$$\theta_{\mathrm{OMC}}=\beta_{0}+\beta_{1}\theta_{\mathrm{MMC}}+\beta_{2}\mathrm{BMI}$$

The generalizability of these models was assessed using leave-one-subject-out cross-validation (LOSO-CV) (28). In this framework, the correction model was iteratively trained on a dataset of 29 participants and validated on the remaining participants; the process was repeated 30 times so that each participant served as the test data once. For a comprehensive performance evaluation, a dataset of approximately 174,000 data points (29 participants × 10 repetitions × 6 s × 100 Hz) was used to capture subtle movement variations.

### 2.5 Statistical analyses

All data processing and statistical computations were performed using MATLAB R2025b (MathWorks, Natick, MA, USA) and Microsoft Excel (Microsoft 365, Redmond, WA, USA). To quantify the estimation accuracy of the uncorrected markerless data and proposed correction models, a full time-series dataset of approximately 174,000 data points was used. The performance was evaluated based on three criteria: 1) root mean square error (RMSE), which penalizes larger deviations more severely; 2) coefficient of determination (*R^2^*), to evaluate the absolute agreement between the MMC models and reference OMC data; and 3) mean bias and 95% limits of agreement (LoA), according to the Bland–Altman method. For statistical comparisons and cohort-level summaries of RMSE and *R^2^*, statistical calculations were performed using mean values per participant (*N* = 30) to account for the inter-individual variability. In contrast, the mean bias, LoA, and proportional bias (*r*) for the Bland–Altman analysis were calculated using the full time-series dataset of approximately 174,000 data points to comprehensively characterize the system's systematic error structure. For all datasets, including the uncorrected MMC data and those from the three correction models (Linear Model, Linear + Anthro Model, and Linear + BMI Model), *R*^2^ was calculated relative to the identity line (*y* = *x*) to assess absolute agreement between the observed and predicted values. Under this definition, *R*^2^ directly quantifies the model's displacement from the gold standard and can yield negative values if the systematic bias or error exceeds the variance of the reference data. To compare RMSE across models, a one-way repeated measures analysis of variance (ANOVA) was performed. *post-hoc* comparisons with Bonferroni corrections were subsequently applied to all pair-wise combinations. Within this analysis, two key comparisons were of particular interest: 1) the correction effect (comparing uncorrected vs. linear corrected models) to determine the impact of time-series-based correction; and 2) the influence of anthropometrics (comparing the linear corrected model vs. + Anthro and + BMI models) to assess the incremental predictive power of body composition features.

Regarding the Bland–Altman analysis, fixed bias was assessed by comparing the mean bias to zero using a one-sample *t*-test. To detect the presence of proportional bias, where estimation error varies depending on the magnitude of the measurement, the Pearson product-moment correlation coefficient (*r*) between the mean values and differences (residuals) in the Bland–Altman plots was calculated. The *r* value indicates the structure of the error, which is distinct from the model prediction accuracy (*R*^2^).

To further characterize the linear relationship between the MMC and OMC data, linear regression analyses were performed for both the uncorrected MMC data and the corrected estimates. The regression slopes and *y*-intercepts were calculated and compared against their ideal values (slope = 1; intercept = 0) using one-sample *t*-tests. These analyses were used to objectively quantify the magnitude of the systematic scaling and offset bias in the raw MMC output and to evaluate the degree of recovery achieved by the correction models.

The statistical significance level was set at *p* < 0.05 for all tests. Data are presented as mean ± standard deviation unless otherwise specified.

## 3 Results

### 3.1 Systematic error in Raw movement output

**Figure 2:**
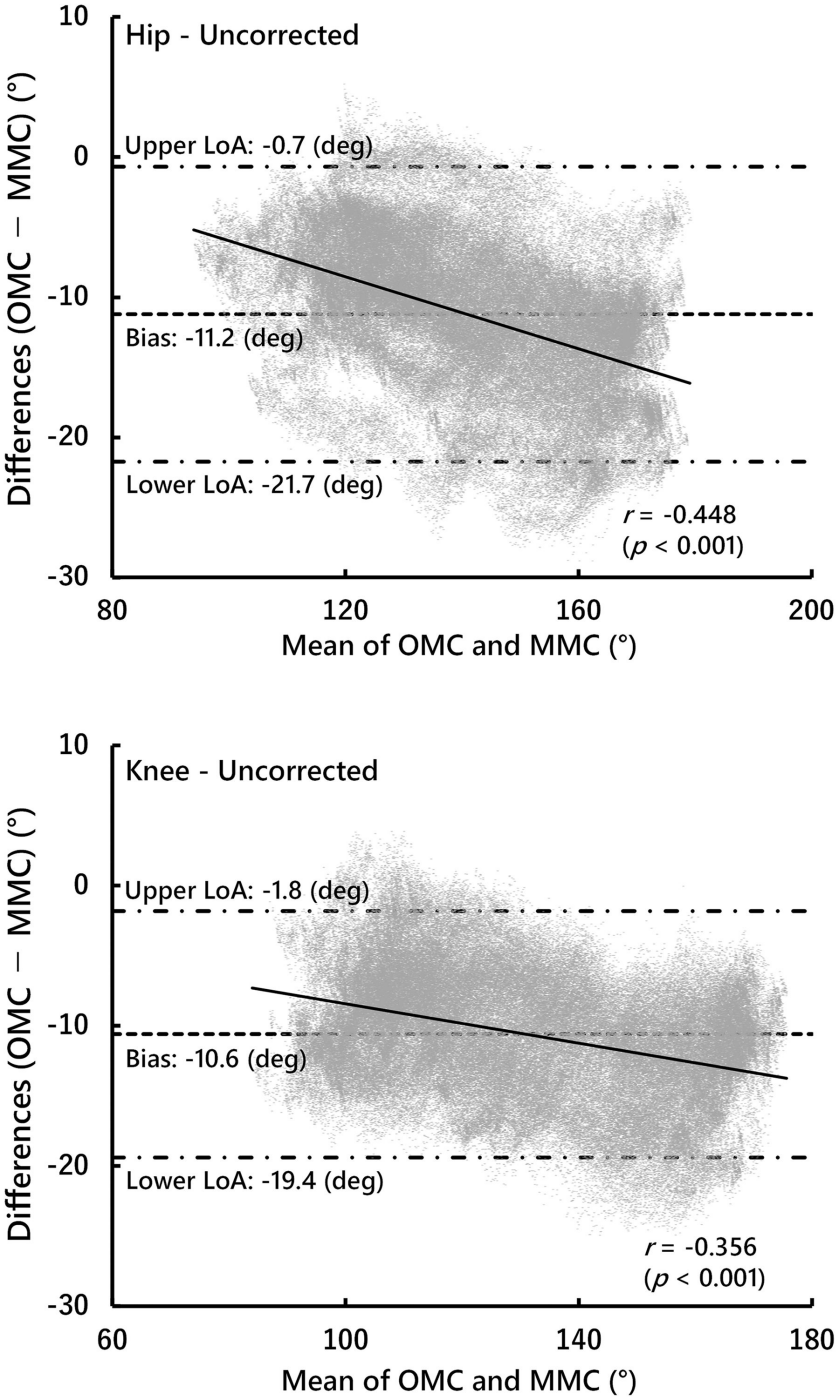
Bland–altman plots comparing markerless motion capture (MMC) system and three-dimensional marker-based optical motion capture (OMC) system for the hip (top) and knee (bottom) joints before correction (*N* = 30). Solid horizontal line indicates the mean bias, and dashed horizontal lines represent the 95% limits of agreement (LoA: bias ± 1.96 standard deviations of the differences).

Prior to correction, the Bland–Altman analysis based on the full time-series dataset (Figure 2) quantified this bias as −11.2° for the hip and −10.6° for the knee (*p* < 0.001 in both cases). Significant proportional errors were also observed, as indicated by the negative regression slopes (hip: *r* = −0.448, *p* < 0.001; knee: *r* = −0.356, *p* < 0.001), confirming that underestimation intensified as the joint angle increased toward full extension.

**Figure 3:**
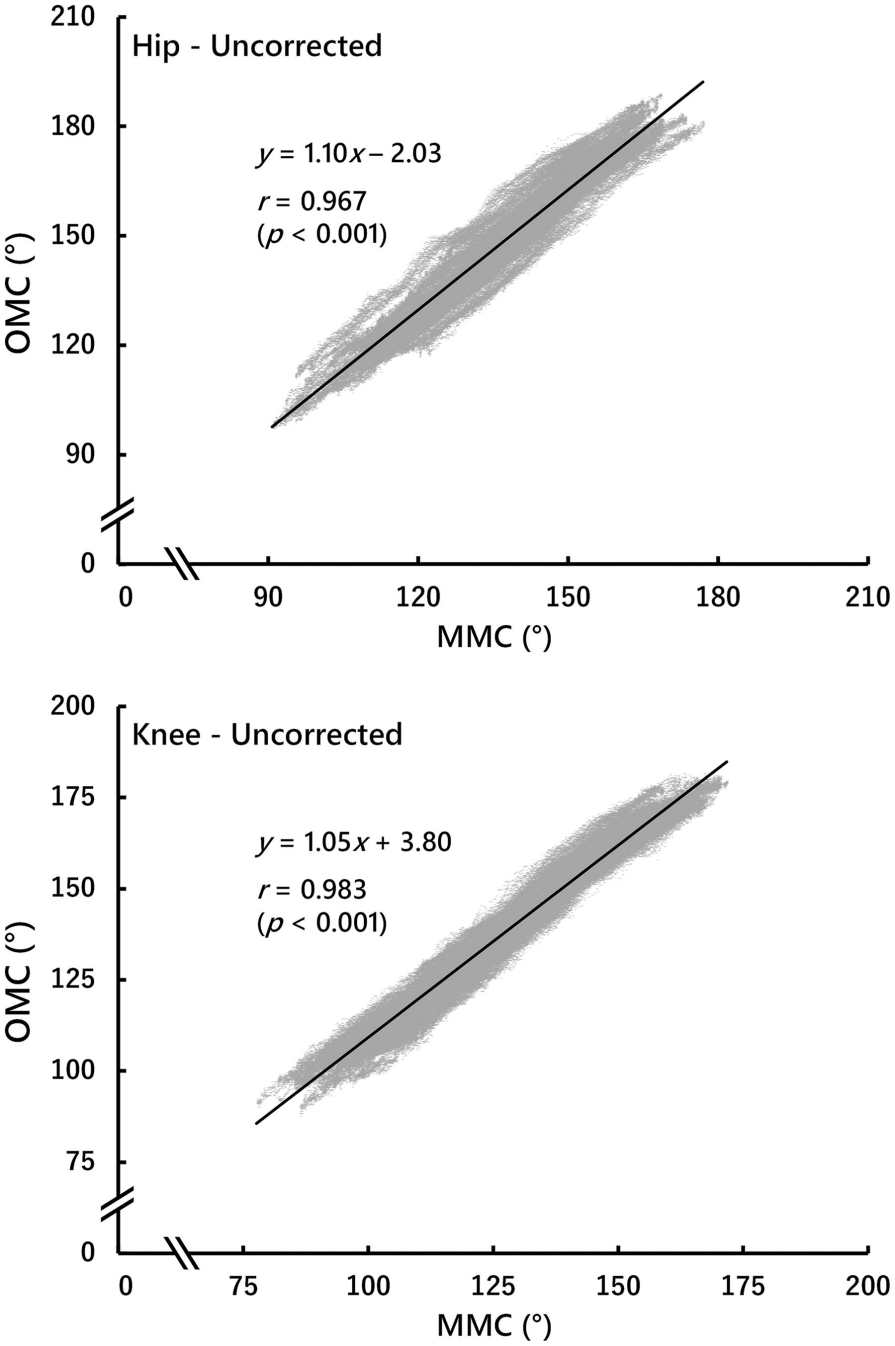
Correlations between the markerless motion capture (MMC) system and the three-dimensional marker-based optical motion capture (OMC) system for the hip (top) and knee (bottom) joints before correction (*N* = 30). All regression slopes differed significantly from 1, and all y-intercepts differed significantly from 0 (all *p* < 0.001).

Correlation analyses further demonstrated systematic deviations from the reference measurements. Despite strong correlations between the raw MMC and OMC data (hip: *r* = 0.967, *p* < 0.001; knee: *r* = 0.983, *p* < 0.001), the regression slopes (hip: 1.10; knee: 1.05) significantly differed from 1, and the regression intercepts (hip: −2.03; knee: 3.80) significantly differed from 0 for both joints (all *p* < 0.001) (Figure 3). These results indicate the presence of systematic scaling and offset errors in the uncorrected MMC estimates.

The cohort-level summary (*N* = 30) further reflected these inaccuracies, showing a high mean RMSE (hip: 11.6° ± 4.2°; knee: 10.9° ± 3.3°) and a low *R^2^* (hip: 0.31 ± 0.94; knee: 0.67 ± 0.30).

### 3.2 Efficacy of the proposed linear correction model

**Figure 4:**
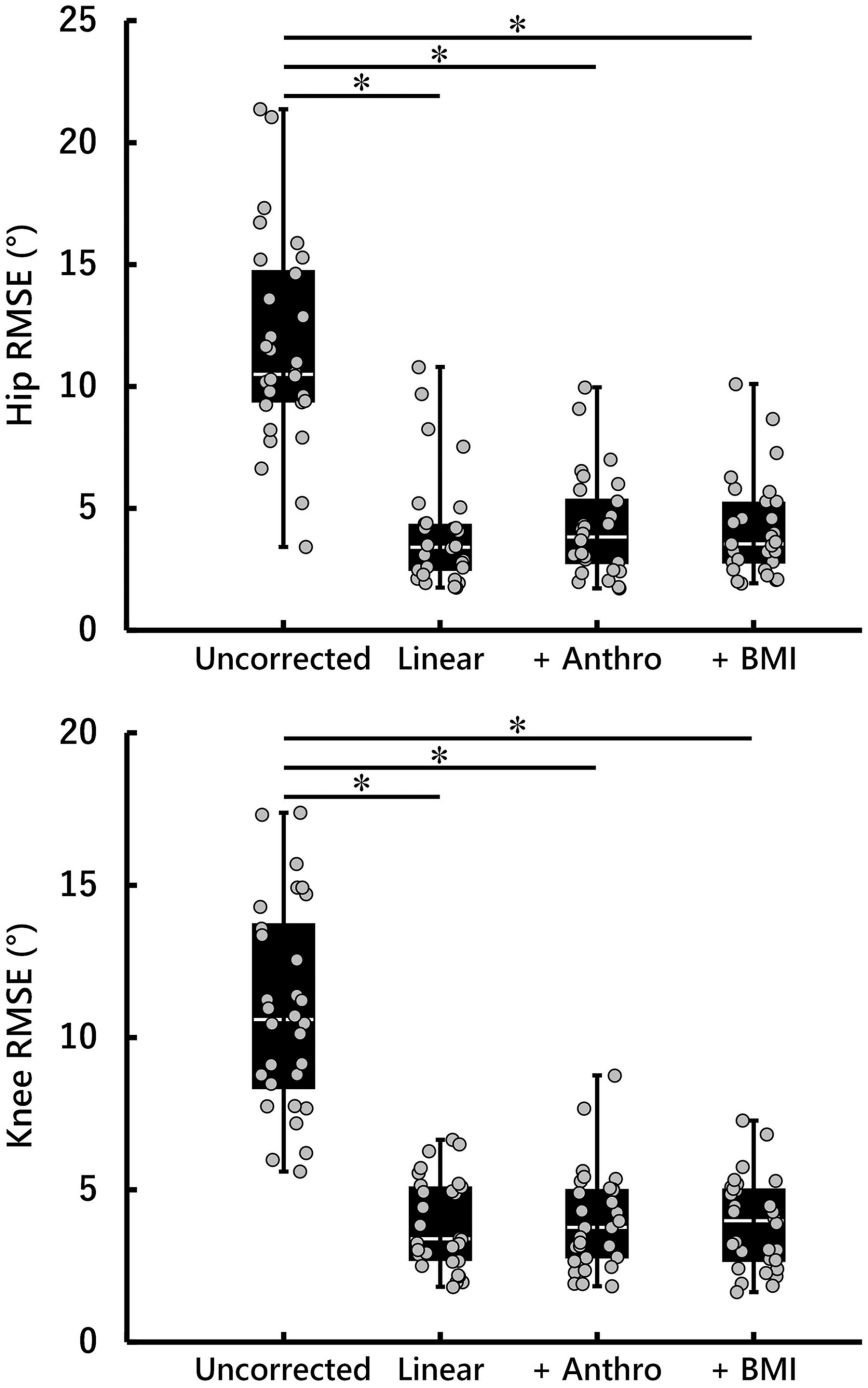
Comparison of root mean square error (RMSE) distributions for the hip (top) and knee (bottom) joints among the four models: uncorrected, linear, + anthro, and + BMI (*N* = 30). Black boxes represent the interquartile range with a horizontal white line indicating the median. Whiskers extend to the maximum and minimum values. The overlying swarm plot displays individual RMSE values for all 30 participants. *: *p* < 0.001.

One-way repeated measures ANOVA revealed a significant main effect of the correction models on RMSE for both the hip (*F*(3, 87) = 90.42, *p* < 0.001) and knee (*F*(3, 87) = 125.01, *p* < 0.001). *post-hoc* comparisons showed that applying the simple linear correction model resulted in a substantial reduction in the RMSE compared with the uncorrected model (hip: 4.0° ± 2.3° vs. 11.6° ± 4.2°; knee: 3.9° ± 1.5° vs. 10.9° ± 3.3°; *p* < 0.001 in both cases) (Figure 4). Furthermore, the goodness-of-fit was further supported by high *R^2^*, reaching 0.90 ± 0.17 for the hip and 0.96 ± 0.04 for the knee.

**Figure 5:**
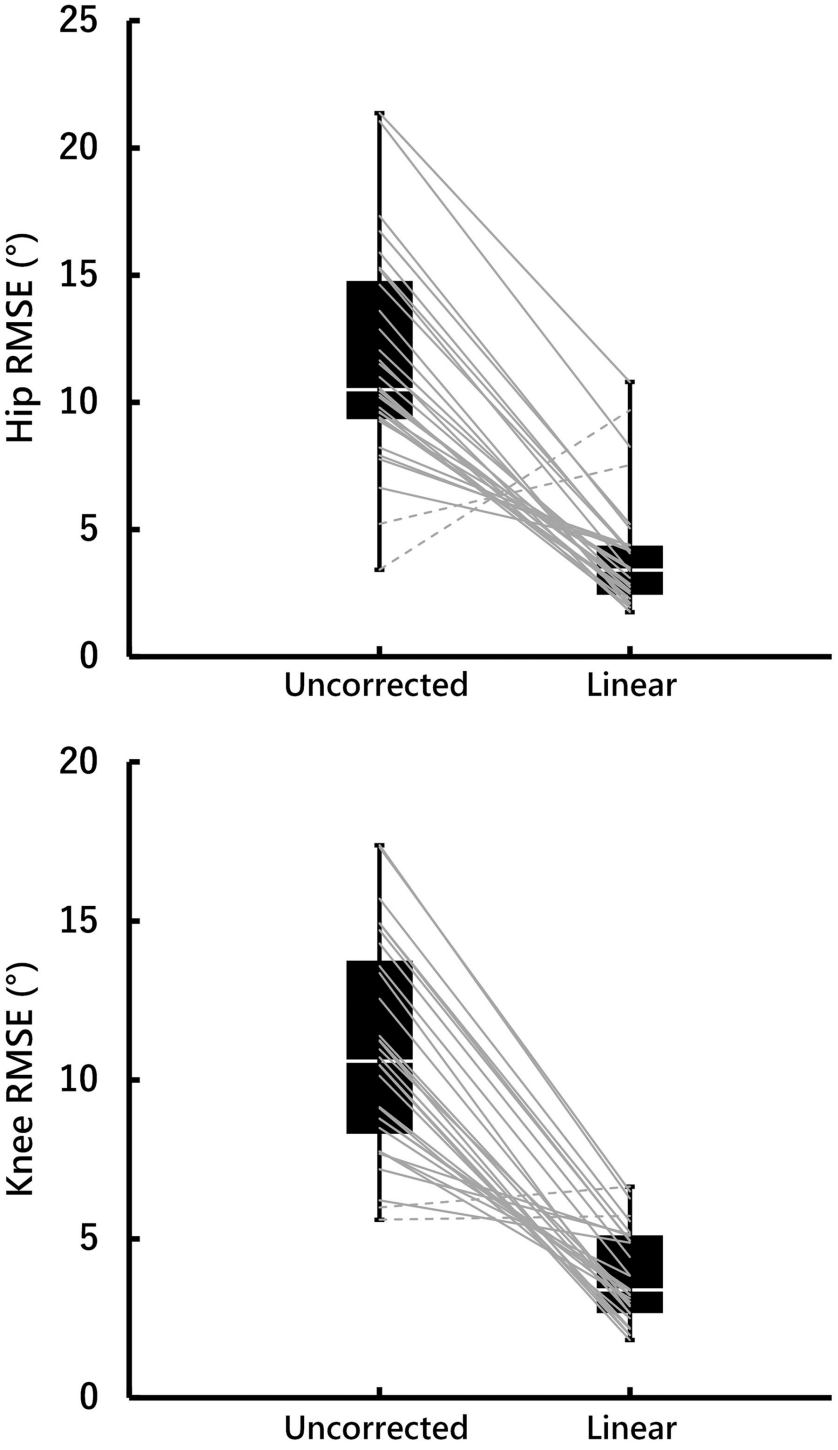
Paired line plots of individual joint angle root mean square error (RMSE) before and after linear correction (*N* = 30). Black boxes represent the interquartile range with a horizontal white line indicating the median. Whiskers extend to the maximum and minimum values. Each gray line represents a single participant; solid lines indicate participants who showed a reduction in the RMSE (improved accuracy), while dashed lines represent those who experienced an increase in the RMSE after correction.

**Figure 6:**
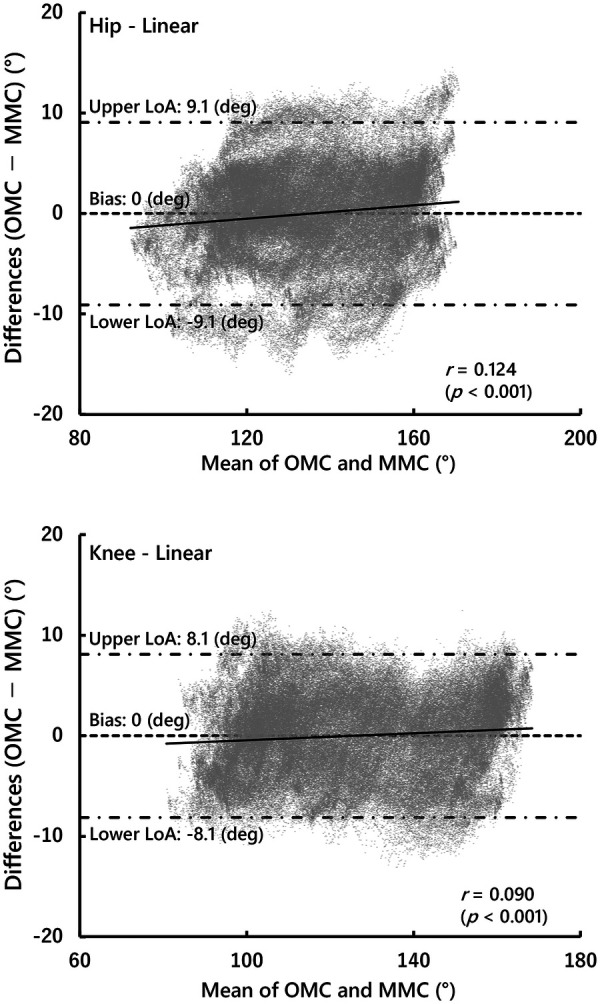
Bland–altman plots comparing markerless motion capture (MMC) system and three-dimensional marker-based optical motion capture (OMC) system for the hip (top) and knee (bottom) joints after linear correction (*N* = 30). Solid horizontal line indicates the mean bias, and dashed horizontal lines represent the 95% limits of agreement (LoA: bias ± 1.96 standard deviations of the differences).

The robustness and generalizability of the framework were demonstrated by individual performance gains, as shown in the paired line plots (Figure 5); post-correction RMSE decreased for 28 out of 30 participants (93.3%) for both the hip and knee joints. Notably, the remaining individuals who did not show reduction already exhibited the lowest initial errors within the cohort (pre-correction RMSE: hip, 3.4° and 5.2°; knee, 5.6° and 6.0°), indicating a ceiling effect where further refinement was limited. This indicates consistent efficacy across diverse movement patterns, particularly for correcting substantial systematic deviations. The post-correction Bland–Altman analysis (Figure 6) confirmed the statistical elimination of systematic offsets, with a mean bias converging to 0° for both joints (hip: *p* = 0.323; knee: *p* = 0.645). Furthermore, Pearson's correlation coefficients (*r*) for the residuals, which indicate proportional bias, showed marked reduction to negligible levels (hip: *r* = 0.124, *p* < 0.001; knee: *r* = 0.090, *p* < 0.001). Although these correlations remained statistically significant because of the high degrees of freedom owing to the large sample size, the negligible magnitude of the coefficients confirmed that depth-dependent errors were effectively mitigated throughout the entire range of motion.

**Figure 7:**
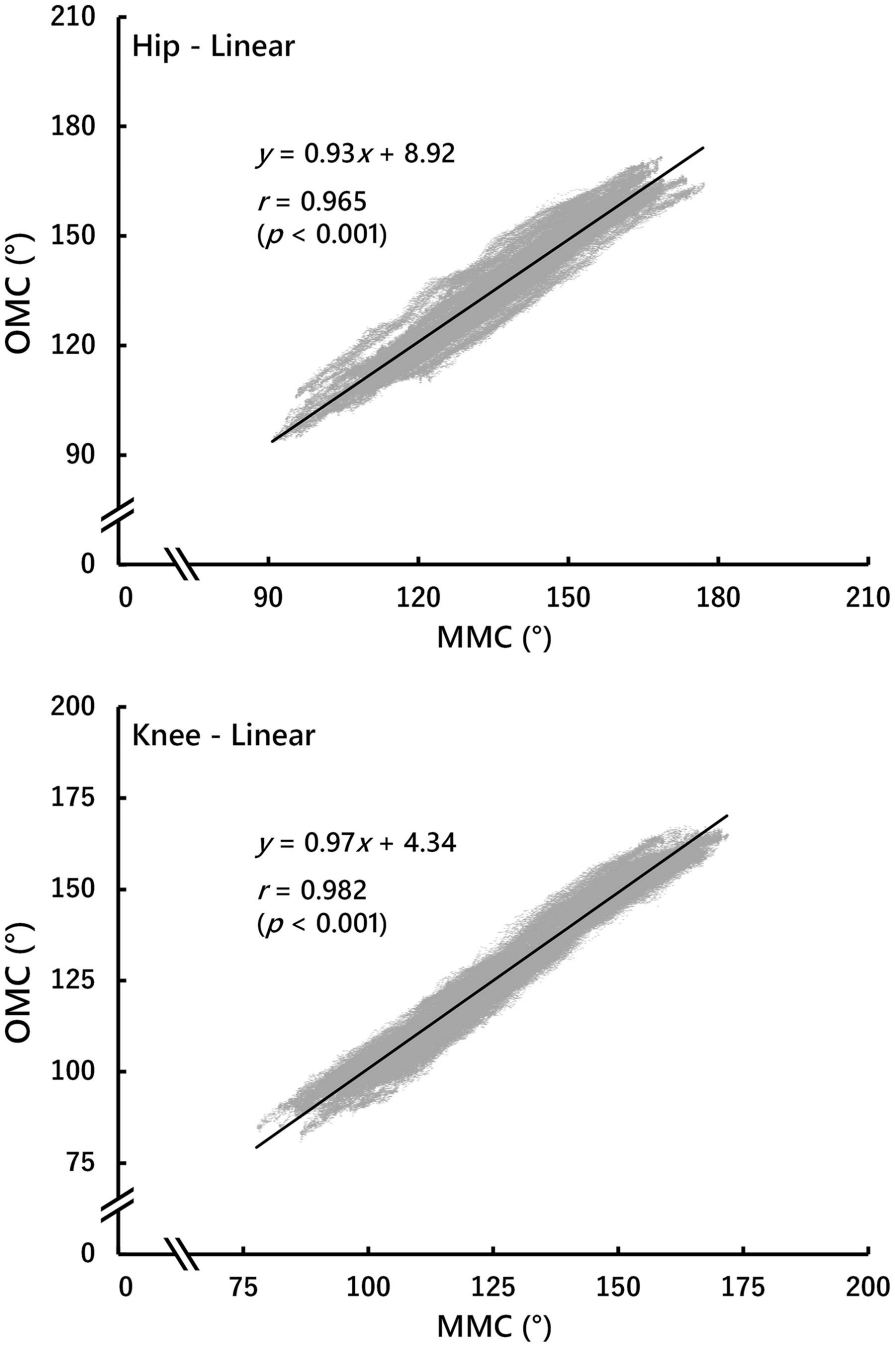
Correlations between the markerless motion capture (MMC) system and the three-dimensional marker-based optical motion capture (OMC) system for the hip (top) and knee (bottom) joints after linear correction (*N* = 30). All regression slopes differed significantly from 1, and all y-intercepts differed significantly from 0 (all *p* < 0.001).

Correlation analyses between the corrected MMC and reference OMC measurements were additionally performed to visualize the relationship between the two measurement systems. Although strong correlations remained after correction (hip: *r* = 0.965, *p* < 0.001; knee: *r* = 0.982, *p* < 0.001), the regression slopes (hip: 0.93; knee: 0.97) remained significantly different from 1, and the intercepts (hip: 8.92; knee: 4.34) significantly differed from 0 (all *p* < 0.001) (Figure 7). These results indicate that the corrected MMC estimates did not achieve a perfect one-to-one correspondence with the reference measurements.

In addition, individual residual errors persisted, with post-correction RMSE ranging from 1.8° to 10.8° for the hip and 1.8° to 6.6° for the knee, reflecting the inherent variability in the markerless tracking performance across participants.

### 3.3 Comparative analysis of extended multivariate models

While both the + Anthro (hip RMSE: 4.2° ± 2.1°; knee RMSE: 4.0° ± 1.6°) and + BMI (hip RMSE: 4.1° ± 2.0°; knee RMSE: 3.9° ± 1.5°) models significantly improved the estimation accuracy relative to the uncorrected data (for all, *p* < 0.001), they did not provide incremental benefits beyond the simple linear correction (for all, *p* = 1.000) (Figure 4). Similarly, no substantial changes were observed in the *R^2^* for both + Anthro (hip: 0.91 ± 0.11; knee: 0.95 ± 0.04) and + BMI (hip: 0.91 ± 0.11; knee: 0.96 ± 0.04) models compared to the simple linear model (hip: 0.90 ± 0.17; knee: 0.96 ± 0.04).

Visual analysis of the RMSE distributions across participants (Figure 5) further supported these findings. The simple linear model exhibited a slightly narrower interquartile range compared with the extended models, suggesting that incorporating additional physical variables may introduce unnecessary complexity without improving model's stability or generalizability to unknown participants.

## 4 Discussion

### 4.1 Interpretation of Key findings

In this study, the accuracy of a webcam-based MMC system was evaluated using MoveNet for squat analysis and a linear correction framework was validated under standardized acquisition conditions. The investigation yielded three primary insights.

First, the raw MoveNet estimates consistently underestimated joint angles, with a systematic bias of −11.2° for the hip and −10.6° for the knee (Figure 2). This bias is primarily attributable to projection-induced geometric distortion, with an additional contribution from definitional differences between visual keypoints and anatomical joint centers previously reported in markerless–OMC comparisons (16). Furthermore, the significant negative correlations in the Bland–Altman plots (hip: *r* = −0.448, *p* < 0.001; knee: *r* = −0.356, *p* < 0.001) (Figure 2) indicate that this underestimation intensifies as the joint angles increase toward full extension. This trend suggests that perspective-induced geometric distortion and segment occlusion during the transition between different squat depths significantly impair the 2D-to-3D projection accuracy of monocular cameras (25).

Second, the proposed linear correction model significantly improved the accuracy, reducing the RMSE to 4.0° ± 2.3° for the hip and 3.9° ± 1.5° for the knee (Figures 4, 5, respectively). These values are well within commonly referenced tolerances of approximately 5° (8, 18). Crucially, the systematic underestimation observed in the raw output was effectively neutralized, with the mean bias converging to 0° for both joints (Figure 6). This was accompanied by high coefficients of determination (≥0.90), confirming that the linear model not only corrected the magnitude of the angles but also maintained high fidelity to the original movement trajectories. Notably, including anthropometric features (BMI, height, and weight) did not significantly improve the estimation accuracy (Figure 4). This finding indicates that the dominant error sources are governed by the systematic geometric and algorithmic characteristics rather than the individual body composition, supporting a simple, interpretable model (27) that preserves privacy and eliminates the need for manual user input.

Third, while the global linear model effectively eliminated group-level systematic bias, a significant individual variability in the residual errors (hip RMSE range: 1.8°–10.8°; knee RMSE range: 1.8°–6.6°) suggests that personal movement characteristics and the spatial resolution limits of the 2D feature extractor still influence the estimation accuracy. Notably, although no RMSE reduction was observed for both joints of two participants, these individuals already exhibited high baseline accuracy (RMSE ≤ 6.0°) prior to correction (Figure 5). This suggests that the linear correction framework effectively targets systematic offsets without introducing artifacts into already precise estimates, thereby maintaining the integrity of high-quality raw captures while mitigating substantial errors in the rest of the cohort.

### 4.2 Nature of systematic pose estimation error

The raw markerless output exhibited substantial estimation errors, with a cohort-level mean RMSE of approximately 11° (hip: 11.6° ± 4.2°; knee: 10.9° ± 3.3°). Combined with low mean *R*^2^ values and high standard deviations relative to the identity line (hip: 0.31 ± 0.94; knee: 0.67 ± 0.30), these metrics reflected a poor initial absolute agreement. These inaccuracies are primarily attributable to the perspective distortion inherent in 2D projections (24). The statistical profile of the raw output was characterized by a significant mean bias (hip: −11.2°; knee: −10.6°) (Figure 2) and low *R*^2^ relative to the identity line. Combined with the proportional errors identified in the Bland–Altman analysis (hip: *r* = −0.448; knee: *r* = −0.356) (Figure 2), as well as the regression slopes (1.05–1.10) and intercepts (−2.03–3.80) that deviated significantly from the identity line (Figure 3), these findings clarify that despite individual variability in the accuracy, overall, MoveNet captures kinematic trajectories with high linearity but suffers from a significant fixed scaling and offset bias. This inherent linearity in the error structure suggests that the systematic deviations are largely predictable, providing a robust theoretical justification for the efficacy of a simple linear correction approach over more complex nonlinear alternatives. This offset reflects a fundamental mismatch between visual keypoints (surface landmarks) and anatomical joint centers (16). Because the markerless algorithm applies these learned rules consistently across individuals, the resulting error is systematic rather than random (7, 16, 29). Similar systematic deviation patterns have been reported in other anatomical regions and movement tasks, suggesting that these projection-related offsets are a common characteristic of monocular markerless systems (30–32).

### 4.3 Morphological independence and practical utility

Notably, augmenting the model with individual anthropometric features (height, weight, and BMI) yielded no improvement in the RMSE (*p* = 1.000) (Figure 4). This morphological independence suggests that the dominant error sources are linked to common algorithmic and optical projection characteristics rather than the individual body composition (25, 29). This finding is significant for real-time browser-based applications using TensorFlow.js, validating a system design that eliminates the need for manual user data input (15, 21). The ability to perform automated landmark identification without user interaction streamlines the assessment process, potentially reducing barriers in conventional 3D motion analysis (14). This not only preserves user privacy through client-side local execution but also maximizes the utility of the system for remote home monitoring where professional assistance for measurement is unavailable (14, 21).

Furthermore, although advanced nonlinear models or subject-specific calibration could potentially reduce residual errors, their integration into browser-based environments often results in significant computational overhead, compromising the real-time nature of the visual feedback (typically >30 fps as specified in our system design). Home-based monitoring must prioritize providing immediate, low-latency guidance through an accessible web interface. Our simple linear model offers an optimal balance between interpretability, robustness, and computational efficiency, enabling a zero-install solution that operates effectively on standard consumer devices without the need for high-performance GPUs.

### 4.4 Individual variability and limitations

The core novelty of this study lies in providing a highly accessible, browser-based monitoring system for home-based exercise. To ensure reliable and reproducible kinematic assessment in such unconstrained environments, we intentionally employed a standardized acquisition geometry. Although fixing the camera distance (2.00 m) and height (0.975 m) limits the system's generalizability, this strategic choice was necessary to minimize unpredictable perspective distortion and ensure data reproducibility. Consequently, the derived linear correction model implicitly incorporates the specific geometric characteristics of this setup. Perspective distortion is inherently sensitive to the ratio between the subject's dimensions and their distance from the optical center (17, 24). Although our fixed setup ensures high internal validity, the generalizability of the correction coefficients to different camera placements remains to be verified. As noted in gait analysis studies, changes in participant's position relative to the camera can alter kinematic estimates (11). Future work should investigate whether a dynamic scaling factor based on the real-time subject-camera distance, potentially derived from MoveNet's bounding box, can further enhance the model's robustness in unconstrained environments.

Another limitation with respect to external validity is that the study population was restricted to healthy young males. Variations in body composition, sex-specific morphological characteristics, and clothing patterns are known to influence the landmark identification accuracy of pose estimation algorithms (33). This study serves as a proof-of-concept for integrated system development, and a more diverse cohort, including females, older adults, and clinical populations, must be considered in future work to rigorously verify the generalizability and robustness of the correction framework across different demographic groups.

Despite the high cohort-level accuracy achieved through the linear correction framework, individual residual errors exhibited subtle fluctuations throughout the squatting range. Regression analyses of the corrected data further revealed that while the systematic offsets were statistically eliminated, the regression slopes (0.93–0.97) and intercepts (4.34–8.92) still deviated significantly from a perfect one-to-one correspondence (Figure 7). These persistent discrepancies indicate that the corrected MMC estimates are not yet identical to the gold-standard OMC measurements at the individual level. These persistent discrepancies are likely attributable to the spatial resolution limits of the 2D feature extractor and phase-dependent keypoint jitter. In markerless systems, these residuals reflect the dynamic mismatch between the visual surface features tracked by the deep learning model and the underlying anatomical joint centers (12, 29).

Although marker-based systems are also affected by soft tissue displacement (34, 35), monocular MMC systems appear to be primarily limited by the precision of visual landmark identification during dynamic movement. Dynamic changes in surface appearance, such as muscle deformation or subtle segment occlusions during the squat, can alter visual features tracked by the algorithm, manifesting as the individual-specific variability. Similar to the well-documented soft tissue artifacts in marker-based systems (36), these dynamic surface-to-bone mismatches in markerless tracking are highly individual-specific and difficult to eliminate using global linear models. While the system captures temporal patterns with high fidelity, the presence of these individual fluctuations explains the remaining discrepancy from the gold-standard OMC (12, 29). Therefore, achieving the sub-degree accuracy will likely require participant-specific calibration, temporal smoothing filters, or complex biomechanical constraints to explicitly model these surface-to-bone relationships (12, 37). Nevertheless, the framework proposed in this study provides sufficient precision and real-time responsiveness for practical monitoring and movement guidance in home-based settings.

## 5 Conclusion

In this study, a browser-based squat-monitoring system using the MoveNet Lightning model was developed and evaluated against a gold-standard 3D marker-based OMC system. Our findings confirmed that raw monocular MMC showed significant systematic underestimation (bias: hip, −11.2°; knee, −10.6°), primarily due to perspective-induced foreshortening and mismatches between the visual keypoints used in deep learning and the anatomical joint centers defined by biomechanical models.

We demonstrated that a simple linear correction model validated through LOSO-CV effectively mitigated this systematic underestimation, reducing the RMSE to less than 4.0°. This performance falls within commonly referenced tolerances of approximately 5° for biomechanical movement analysis, suggesting practical applicability under standardized acquisition conditions. Notably, the effectiveness of this correction remained independent of individual anthropometric features such as height, weight, and BMI, supporting the system's “plug-and-play” feasibility for diverse populations. This independence, combined with privacy-conscious local execution via TensorFlow.js, supports the feasibility of deploying condition-aware motion analysis tools in home-based rehabilitation and remote monitoring environments.

However, detailed residual analysis revealed that individual errors exhibited subtle fluctuations that could not be fully eliminated by linear models. These discrepancies likely reflect the inherent limits of visual landmark identification, where dynamic surface deformations and keypoint jitter manifest as individual-specific nonlinearities. Future work will focus on 1) implementing a brief personalized calibration phase to accommodate these individual nonlinearities and 2) exploring monocular 3D pose estimation frameworks to increase robustness against extreme perspective distortions and occlusions.

## Data Availability

The raw data supporting the conclusions of this article will be made available by the authors, without undue reservation.
